# Supplementary material for: Gut epithelium modifies enteric behaviors during nutritional adversity via distinct peptidergic signaling axes
Source: Sci Adv. 2025 Sep 24;11(39):eadw1270. doi: 10.1126/sciadv.adw1270 (PMC12459421; doi:10.1126/sciadv.adw1270)
Supplement: Supplementary file 1 — Figs. S1 to S8 Legends for data S1 to S4 [file sciadv.adw1270_sm.pdf]

Science Advances
AAAS

Supplementary Materials for

Gut epithelium modifies enteric behaviors during nutritional adversity via distinct peptidergic signaling axes

Surojit Sural *et al.*

Corresponding author: Surojit Sural, ss5950@columbia.edu; Oliver Hobert, or38@columbia.edu

Sci. Adv. **11**, eadw1270 (2025)
DOI: 10.1126/sciadv.adw1270

The PDF file includes:

Figs. S1 to S8
Legends for data S1 to S4

Other Supplementary Material for this manuscript includes the following:

Data S1 to S4

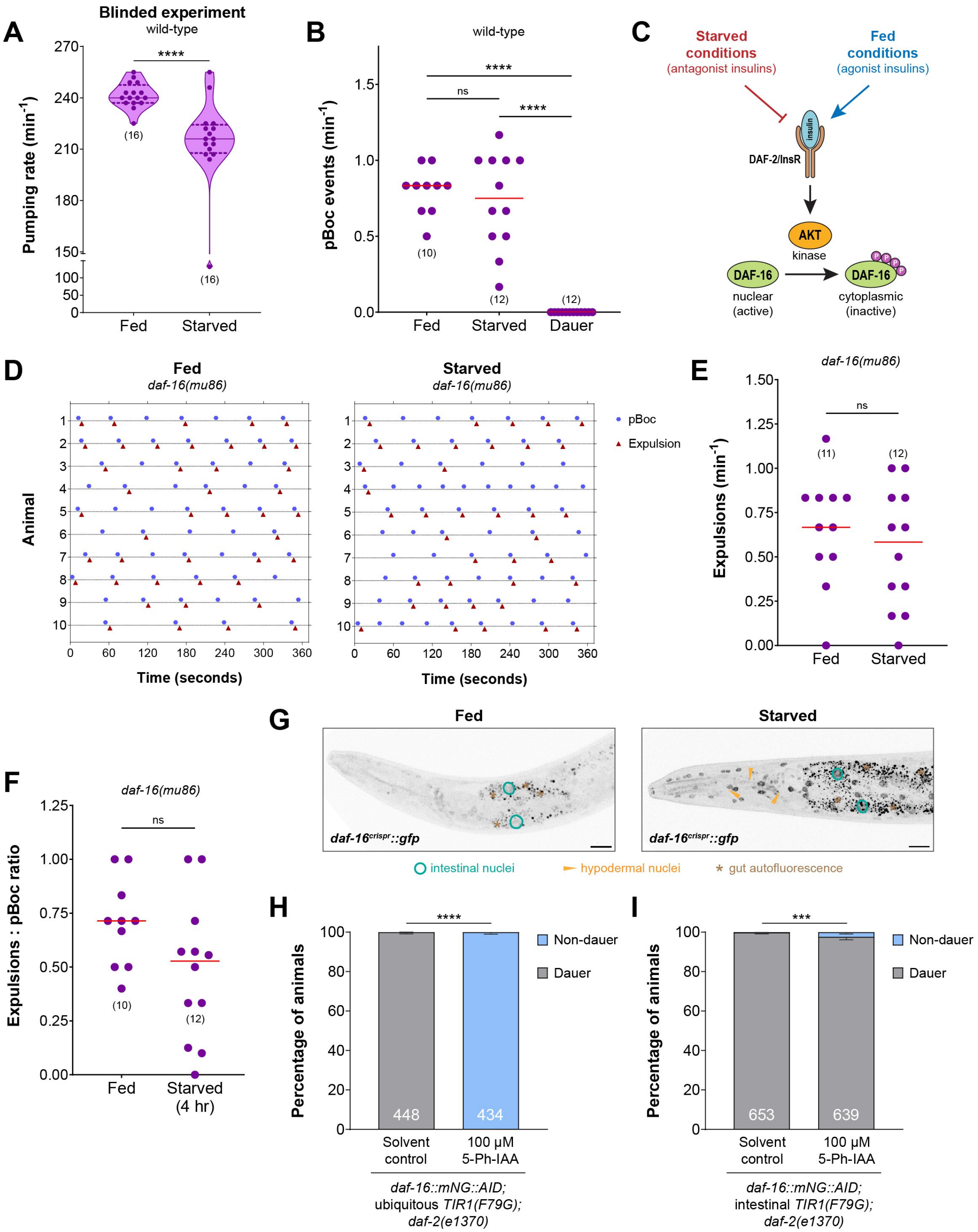

Fig. S1. DAF-16/FoxO is required for starvation-induced inhibition of defecation behvaior.

(A) Pharyngeal pumping rate on food in fed vs starved (4 hr) wild-type adults in a

blinded experiment.

(B) Frequency of pBoc events on food in fed vs starved (4 hr) wild-type adults and in starvation-induced dauer stage animals.

(C) Schematic of DAF-2/InsR signaling regulating DAF-16 nuclear localization in fed vs starved conditions.

(D) Representative traces of pBoc and expulsion events on food in fed vs starved (4 hr) *daf-16(mu86)* adults (10 animals per condition).

(E) Frequency of expulsion on food in fed vs starved (4 hr) *daf-16(mu86)* adults.

(F) Expulsion : pBoc ratio on food in fed vs starved (4 hr) *daf-16(mu86)* adults.

(G) Endogenous DAF-16 protein localization in fed vs starved (4 hr) *daf-16(ot971[daf-16::gfp])* adults. Representative images of 15 animals per condition. Scale bars, 20 µm.

(H) Proportion of SDS-resistant dauers at 25°C in *daf-16(ot853); cshIs140[rps-28p::TIR1(F79G)]; daf-2(e1370)* animals treated with either solvent (ethanol) or 100 µM 5-Ph-IAA.

(I) Proportion of SDS-resistant dauers at 25°C in *daf-16(ot853); otSi2[ges-1p::TIR1(F79G)]; daf-2(e1370)* animals treated with either solvent (ethanol) or 100 µM 5-Ph-IAA.

Horizontal line in the middle of data points represents median value of biological replicates in **(A, B, E, F)**. Additional horizontal lines represent 25th and 75th percentiles in **(A)**. Error bars represent 95% confidence intervals in **(H, I)**. ***, **** and ns represent $P < 0.001$, $P < 0.0001$ and not significant, respectively, in Mann-Whitney test in **(A, E, F)**, in Dunn's multiple comparison test after Kruskal-Wallis test in **(B)** and in Chi-squared test in **(H, I)**.

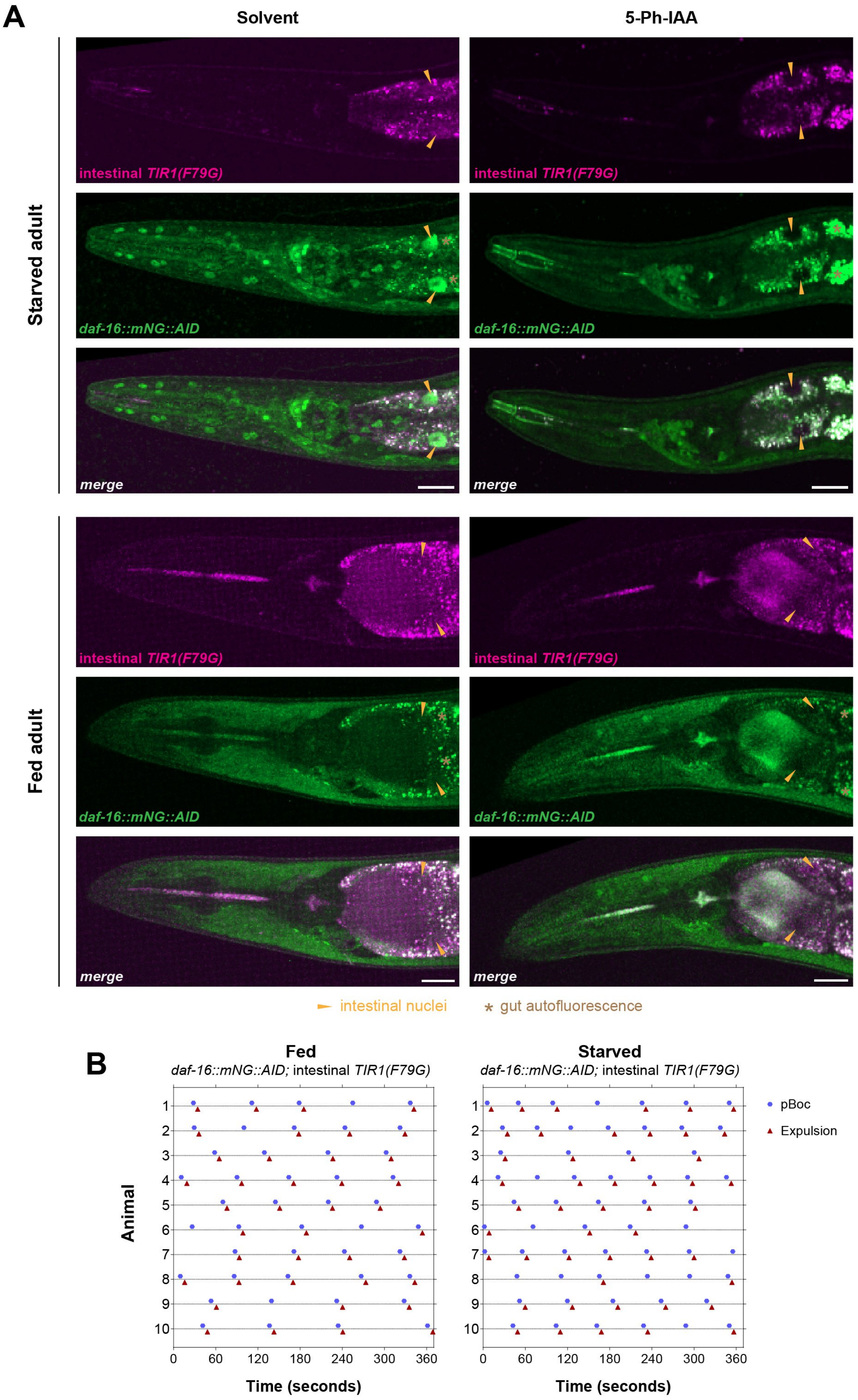
A
Solvent
5-Ph-IAA
Starved adult
Fed adult
intestinal TIR1(F79G)
daf-16::mNG::AID
merge
intestinal nuclei
gut autofluorescence
B
Fed
daf-16::mNG::AID; intestinal TIR1(F79G)
Starved
daf-16::mNG::AID; intestinal TIR1(F79G)
Animal
Time (seconds)
pBoc
Expulsion
0
60
120
180
240
300
360
1
2
3
4
5
6
7
8
9
10

Fig. S2. DAF-16/FoxO activity in the midgut non-cell autonomously regulates defecation behavior.

(A) Intestine-specific DAF-16 depletion in *daf-16(ot853); otSi2[ges-1p::TIR1(F79G)]* starved (4 hr) or fed adults. Animals were treated with either solvent (ethanol) or 100 μM 5-Ph-IAA. DAF-16 was not detected in the midgut epithelial cells after 5-Ph-IAA treatment in 15/15 animals. Scale bars, 20 μm.

(B) Representative traces of pBoc and expulsion events on food after intestine-specific DAF-16 depletion in fed vs starved (4 hr) *daf-16(ot853); otSi2[ges-1p::TIR1(F79G)]* adults treated with 100 μM 5-Ph-IAA (10 animals per condition).

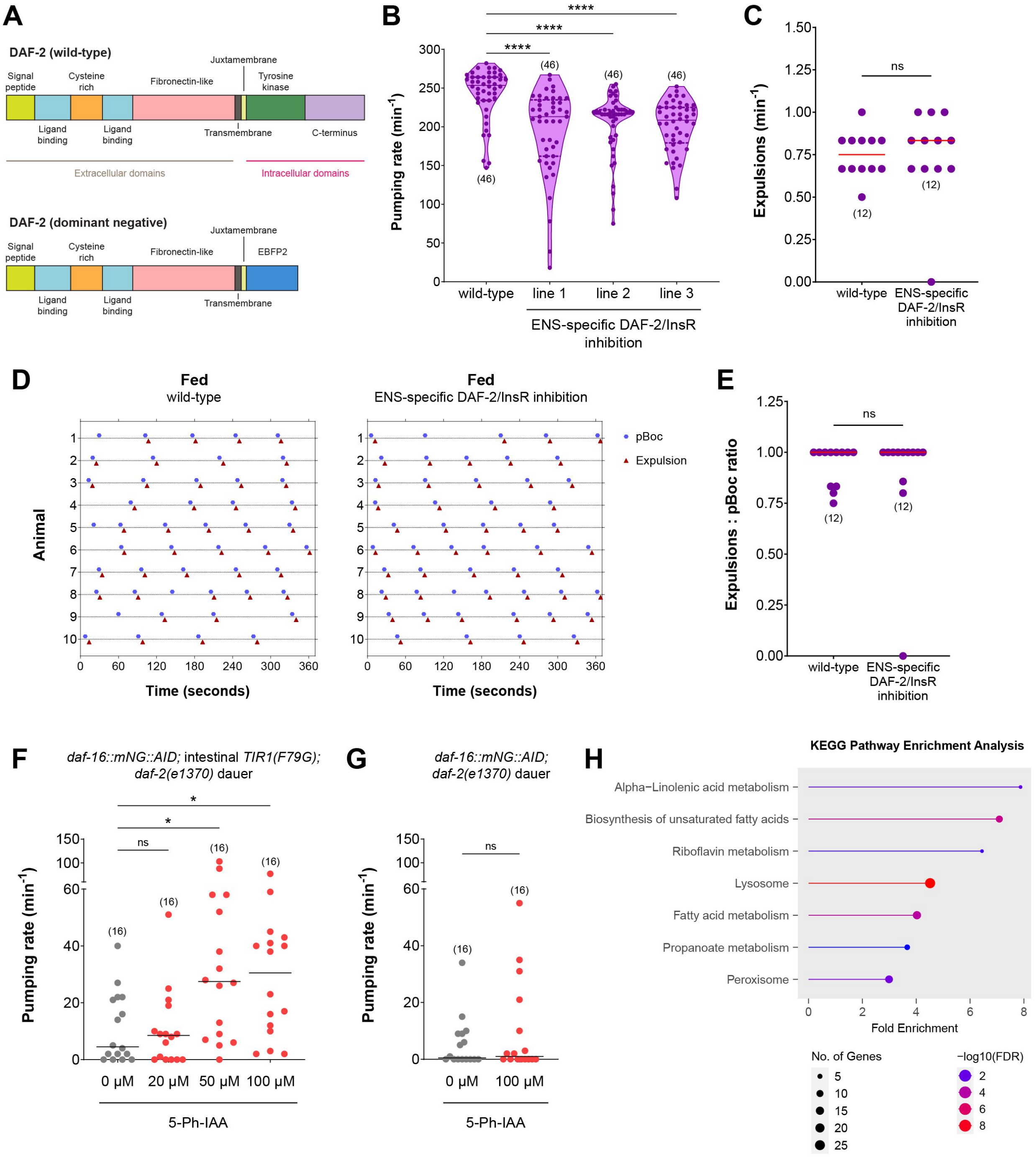

Fig. S3. DAF-16/FoxO activity in the midgut regulates ENS output via modulating gut-to-ENS insulin signaling.

(A) Schematic showing the design of DAF-2(DN) construct in which the intracellular kinase domain of DAF-2/InsR is replaced by EBFP2 fluorescent protein.

(B) Pharyngeal pumping rate on food in fed wild-type and *pha-4prom2::daf-2(DN)*-

expressing (*otIs911*, *otIs912* and *otIs913* lines) adults.

(C) Frequency of expulsion on food in fed wild-type and *otIs913[pha-4prom2::daf-2(DN)]* adults.

(D) Representative traces of pBoc and expulsion (Exp) events on food in fed wild-type and *otIs913[pha-4prom2::daf-2(DN)]* adults (10 animals per condition).

(E) Expulsion : pBoc ratio on food in fed wild-type and *otIs913[pha-4prom2::daf-2(DN)]* adults.

(F) Pharyngeal pumping rate on food in *daf-16(ot853); otSi2[ges-1p::TIR1(F79G)]; daf-2(e1370)* dauer stage animals treated with either solvent (ethanol) or different concentrations of 5-Ph-IAA.

(G) Pharyngeal pumping rate on food in *daf-16(ot853); daf-2(e1370)* dauer stage animals treated with either solvent (ethanol) or 100 µM 5-Ph-IAA.

(H) KEGG pathway enrichment analysis of genes that are upregulated after intestine-specific DAF-16 depletion in dauer stage animals.

Horizontal line in the middle of data points represents median value of biological replicates in **(B, C, E-G)**. Additional horizontal lines represent 25th and 75th percentiles in **(B)**. *, **** and ns represent $P < 0.05$, $P < 0.0001$ and not significant, respectively, in Dunn's multiple comparison test after Kruskal-Wallis test in **(B, F)** and in Mann-Whitney test in **(C, E, G)**.

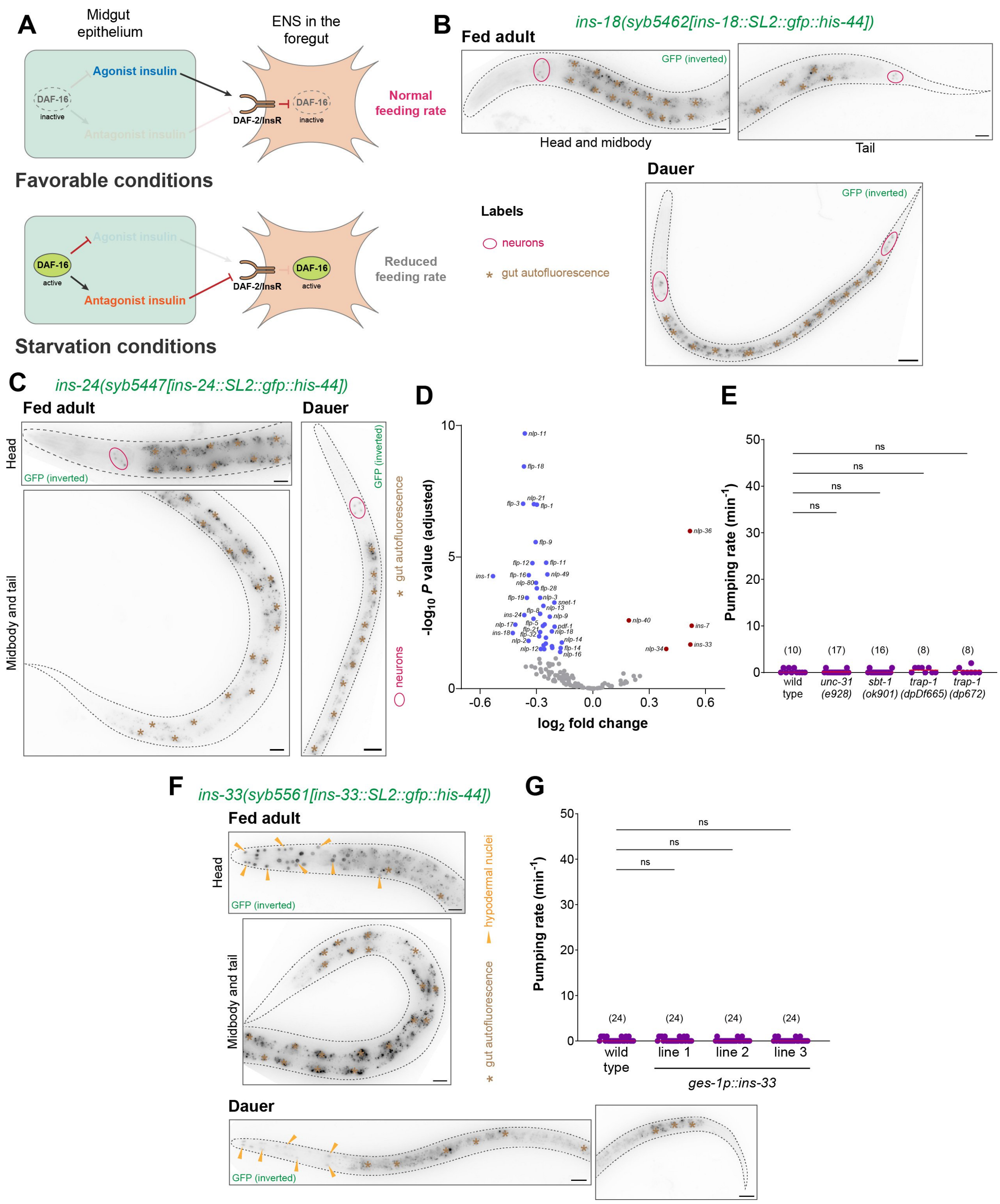

Fig. S4. DAF-16/FoxO activity in the dauer intestine changes the expression of insulin genes both autonomously and non-cell autonomously.

(A) Two alternative models for insulin peptides released from the intestine regulating

output from the ENS in the foregut – (1) absence of agonist insulin or (2) release of antagonist insulin reduces the rate of foregut contractions in starvation conditions.

(B) Expression of the endogenously tagged *ins-18* reporter allele *syb5462[ins-18::SL2::gfp::his-44]* in fed adult and starvation-induced dauer stage animals. Representative images of 15 animals per condition. Scale bars, 20 μm.

(C) Expression of the endogenously tagged *ins-24* reporter allele *syb5447[ins-24::SL2::gfp::his-44]* in fed adult and starvation-induced dauer stage animals. Representative images of 15 animals per condition. Scale bars, 20 μm.

(D) Volcano plot for all neuropeptide family genes that are differentially expressed after intestine-specific DAF-16 depletion in dauers.

(E) Pharyngeal pumping rate on food in wild type, *unc-31(e928)*, *sbt-1(ok901)*, *trap-1(dpDf665)* and *trap-1(dp672)* starvation-induced dauer stage animals.

(F) Expression of the endogenously tagged *ins-33* reporter allele *syb5561[ins-33::SL2::gfp::his-44]* in fed adult and starvation-induced dauer stage animals. Representative images of 15 animals per condition. Scale bars, 20 μm.

(G) Pharyngeal pumping rate on food in starvation-induced dauer stage animals that constitutively express *ins-33* in the intestine (*otEx8326*, *otEx8327* and *otEx8328*). Horizontal line in the middle of data points represents median value of biological replicates in **(E, G)**. ns represents not significant in Dunn's multiple comparison test after Kruskal-Wallis test in **(E, G)**.

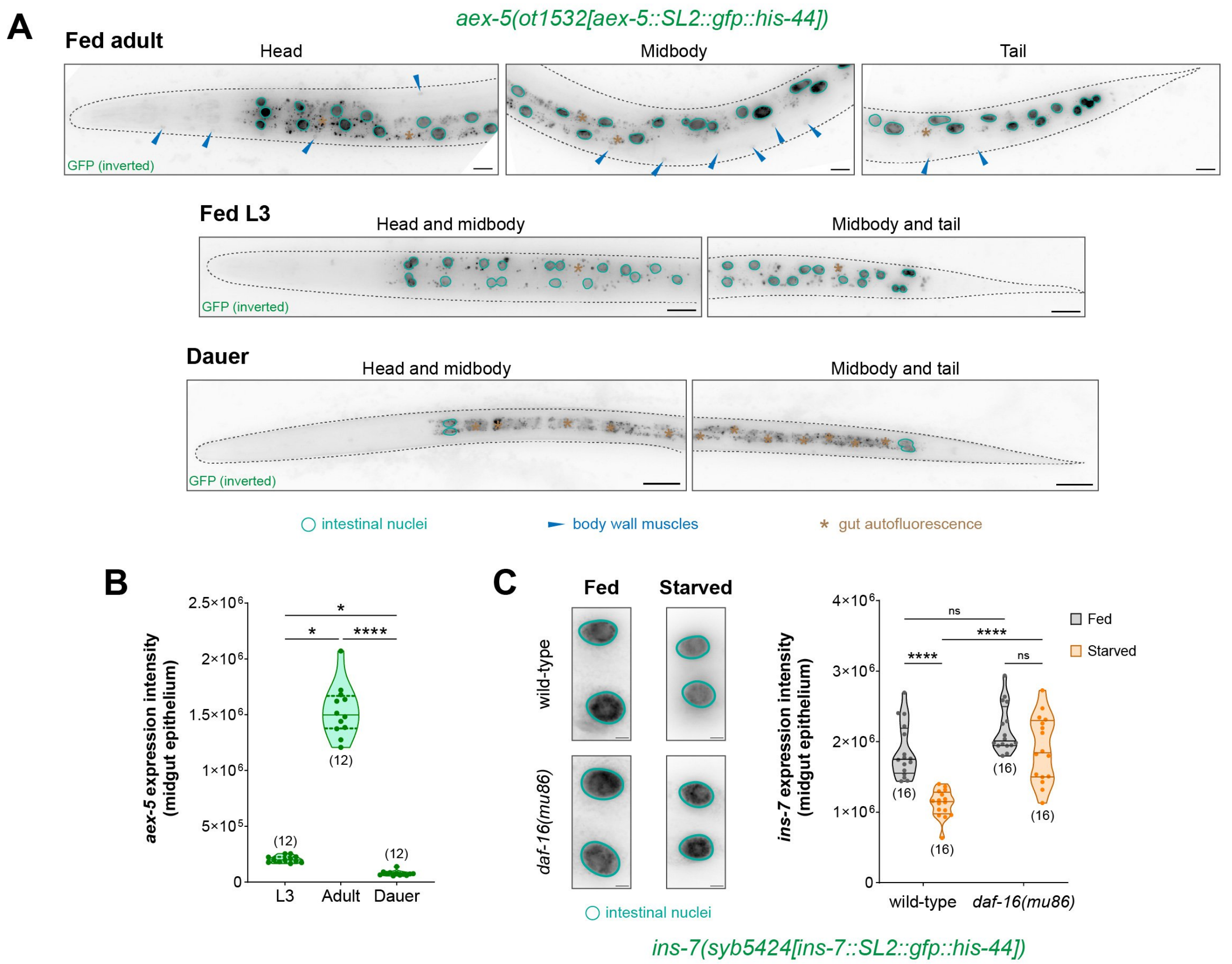

Fig. S5. Prolonged starvation inhibits neuropeptide secretion from the epithelial cells of the midgut.

(A) Expression of the endogenously tagged *aex-5* reporter allele *aex-5(ot1532[aex-5::SL2::gfp::his-44])* in fed adult and L3 larva, and in starvation-induced dauer stage animals.

(B) Quantification of *aex-5* expression in midgut epithelial cells of fed adult and L3 larva, and starvation-induced dauer stage animals.

(C) Expression of the endogenously tagged *ins-7* reporter allele *syb5424[ins-7::SL2::gfp::his-44]* in the midgut epithelial cells of fed vs starved (24 hr) adults.

Horizontal line in the middle of data points and additional horizontal lines represent median of biological replicates, and 25th and 75th percentiles, respectively in **(B, C)**. *, **** and ns represent $P < 0.05$, $P < 0.0001$ and not significant, respectively, in Dunn's

multiple comparison test after Kruskal-Wallis test in **(B)** and in Sidak's multiple comparisons test after two-way ANOVA in **(C)**. Scale bars, 20 μm in **(B)** and 5 μm in **(C)**.

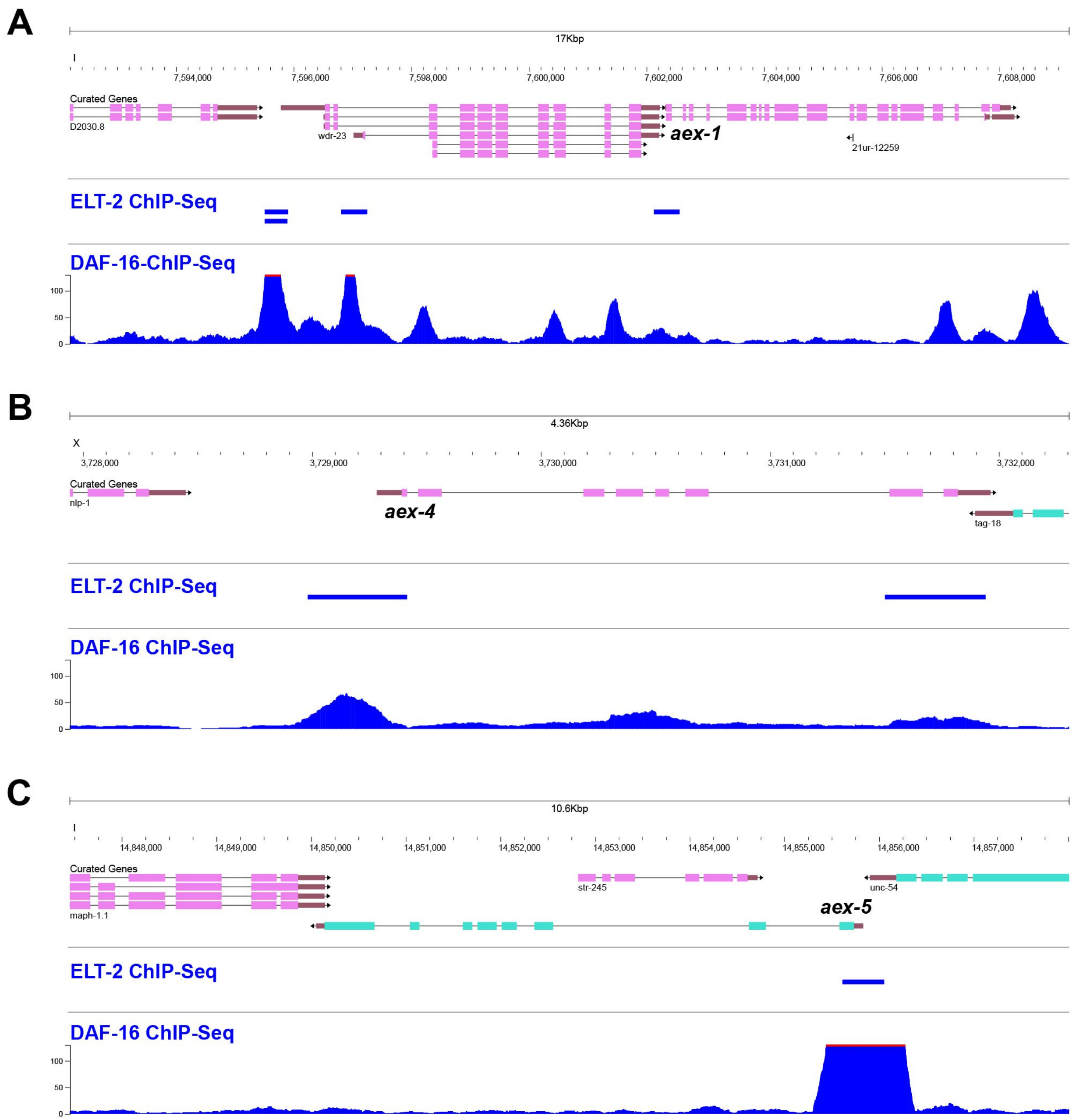

Fig. S6. DAF-16/FoxO and the GATA transcription factor ELT-2 have overlapping binding peaks in the upstream genomic region of *aex* genes.

(A) Location of ChIP-seq peaks for ELT-2 and DAF-16 in the genomic locus of *aex-1*.

(B) Location of ChIP-seq peaks for ELT-2 and DAF-16 in the genomic locus of *aex-4*.

(C) Location of ChIP-seq peaks for ELT-2 and DAF-16 in the genomic locus of *aex-5*.

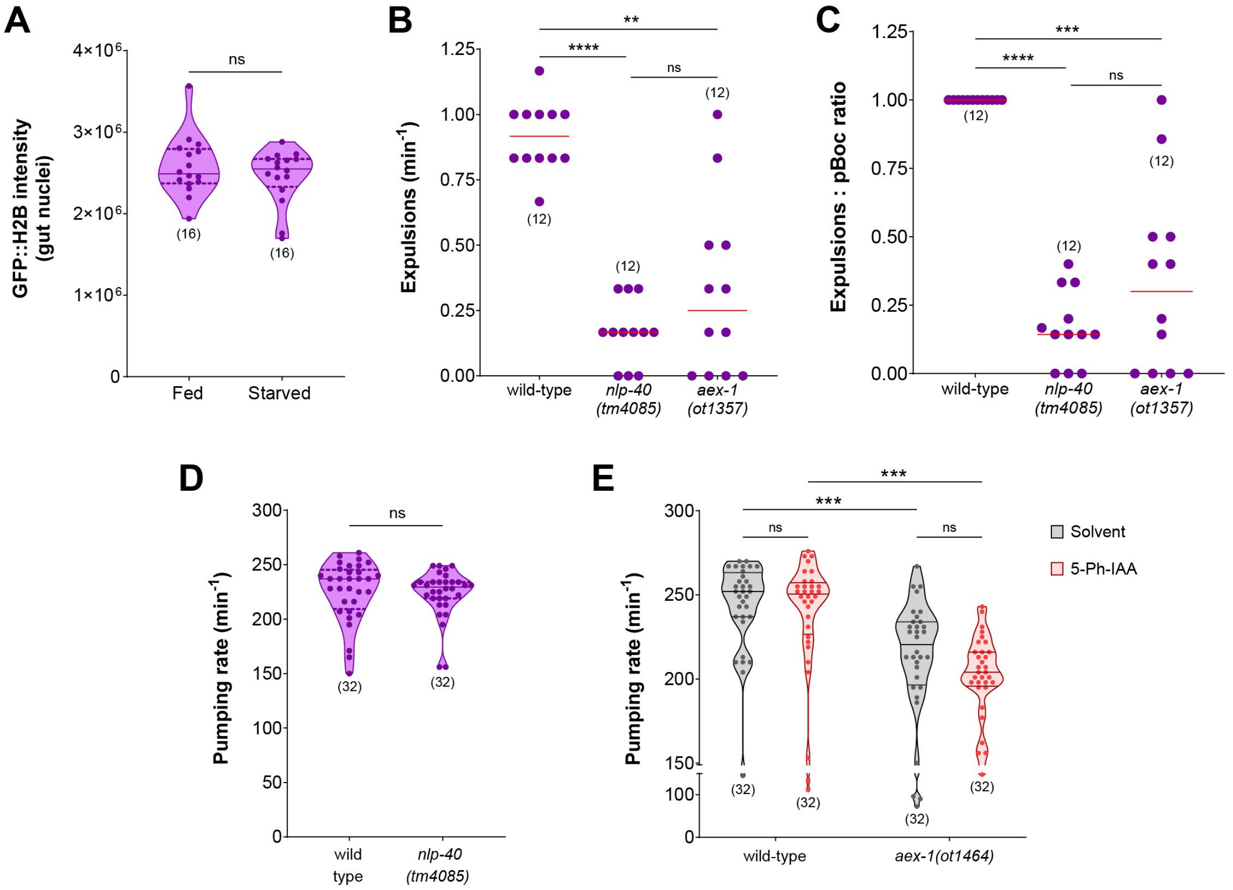

Fig. S7. The rate of defecation, but not feeding, is inhibited in animals lacking the gut-derived neuropeptide NLP-40.

(A) Quantification of GFP::H2B intensity in midgut nuclei of fed vs starved (24 hr) adults expressing *otIs904[ges-1p::ins-1::tagRFP::SL2::gfp::his-44]*.

(B, C) Frequency of expulsion and expulsion : pBoc ratio on food in fed wild-type, *nlp-40(tm4085)* and *aex-1(ot1357)* adults.

(D) Pharyngeal pumping rate on food in fed wild-type and *nlp-40(tm4085)* adults.

(E) Pharyngeal pumping rate on food in fed *daf-16(ot853)* adults without *TIR1(F79G)* in wild-type or *aex-1(ot1464)* genetic background. Animals were treated with either solvent (ethanol) or 100 μM 5-Ph-IAA.

Horizontal line in the middle of data points represents median value of biological replicates in **(A-E)**. Additional horizontal lines represent 25th and 75th percentiles in **(A, D, E)**. **, ***, **** and ns represent $P < 0.01$, $P < 0.001$, $P < 0.0001$ and not significant, respectively, in Mann-Whitney test in **(A, D)**, in Dunn's multiple comparison test after

Kruskal-Wallis test in **(B, C)**, and in Sidak's multiple comparisons test after two-way ANOVA in **(E)**.

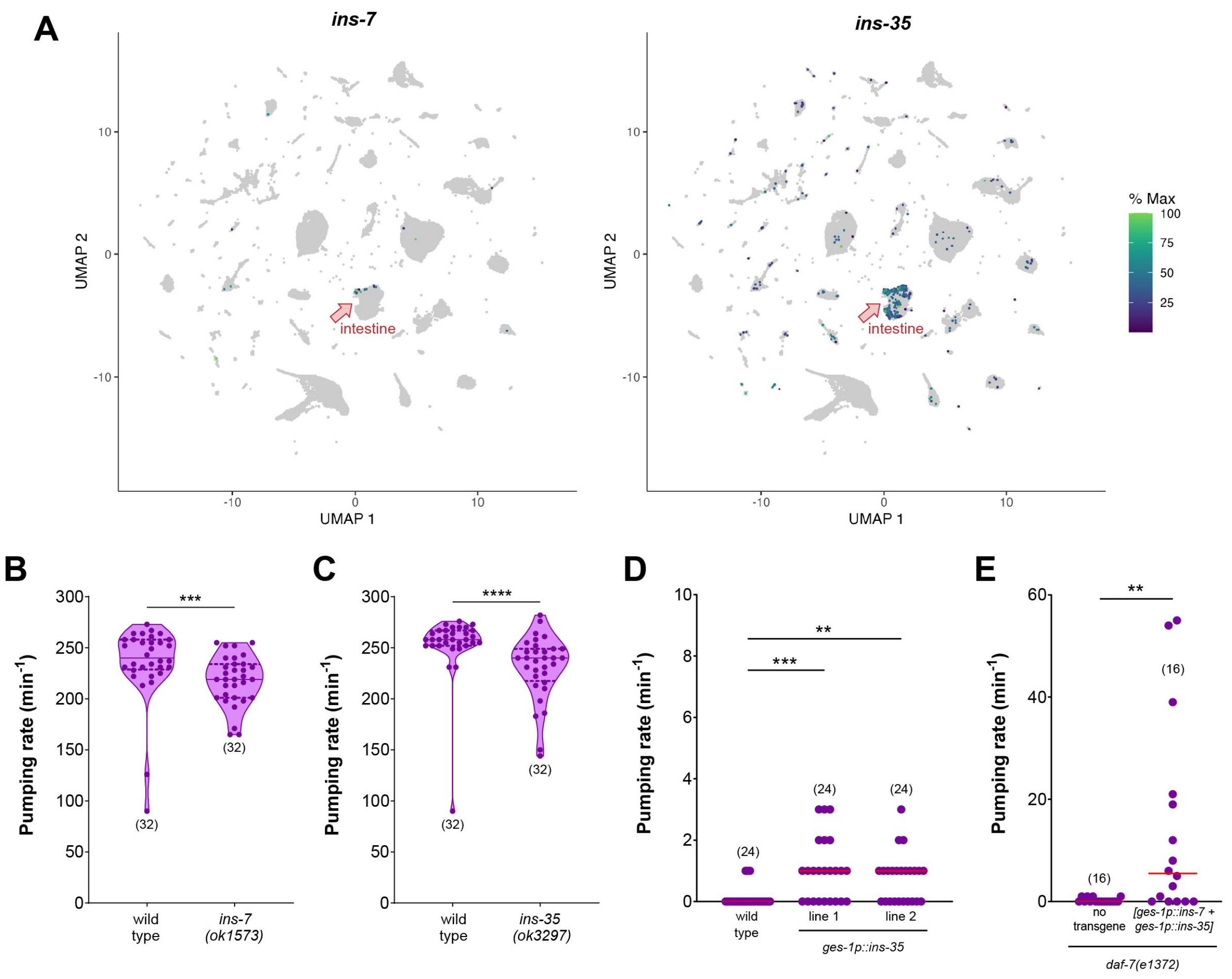

Fig. S8. Secretion of gut-derived insulins is both necessary and sufficient for foregut contractility.

(A) Intestine-specific expression of *ins-7* and *ins-35* retrieved from single-cell RNA-seq data.

(B) Pharyngeal pumping rate on food in fed wild-type and *ins-7(ok1573)* adults.

(C) Pharyngeal pumping rate on food in fed wild-type and *ins-35(ok3297)* adults.

(D) Pharyngeal pumping rate on food in starvation-induced dauer stage animals that constitutively express *ins-35* in the intestine (*otEx8242* and *otEx8243*).

(E) Pharyngeal pumping rate on food in *daf-7(e1372)* dauer stage animals that constitutively co-express *ins-7* and *ins-35* in the intestine (*otEx8223*).

Horizontal line in the middle of data points represents median value of biological replicates in **(B-E)**. Additional horizontal lines represent 25th and 75th percentiles in **(B,**

C). **, *** and **** represent $P < 0.01$, $P < 0.001$ and $P < 0.0001$, respectively, in Mann-Whitney test in **(B, C, E)** and in Dunn's multiple comparison test after Kruskal-Wallis test in **(D)**.

Data S1: Differentially expressed genes after intestinal DAF-16 depletion in dauers. (separate file)

List of all genes that are differentially expressed after intestine-specific DAF-16 removal in dauers using AID2, in comparison to either no TIR1 or no 5-Ph-IAA control.

Data S2: Enrichment of transcription factor binding sites in promoter regions of genes differentially expressed after intestinal DAF-16 depletion in dauers. (separate file)

Data S3: List of *C. elegans* strains used in this study. (separate file)

Data S4: crRNA and single-stranded oligodeoxynucleotide sequences used for CRISPR/Cas9-mediated genome editing. (separate file)
